# Impacted Lower Second Permanent Molars at the Ramus and Coronoid Process: A New Clinical Symptom of the WNT10A Mutation in Ectodermal Dysplasia

**DOI:** 10.5005/jp-journals-10005-1466

**Published:** 2017-02-27

**Authors:** Elia Sfeir, Samia Aboujaoude

**Affiliations:** 1 Professor, Department of Pediatric Dentistry, School of Dentistry Lebanese University, Beirut, Lebanon; 2 Associate Professor, Department of Pediatric Dentistry, School of Dentistry Lebanese University, Beirut, Lebanon

**Keywords:** Ectodermal dysplasia, Hidrotic, Impacted molar, WNT10A mutation.

## Abstract

**Aim:**

Hidrotic ectodermal dysplasia (ED) with the WNT10A mutation produces variable dentofacial symptoms. The aim of this study was to describe a new clinical symptom, i.e., specific to the WNT10A mutation in hidrotic ED. The study investigated the migratory trend of the lower second permanent molars to the ramus or coronoid process. To the best of authors’ knowledge, no data in the literature describe this trend in cases of hidrotic ED.

**Materials and methods:**

A three-generation family pedigree was established for seven families after the diagnosis of hidrotic ED in a 10-year-old boy. Thereafter, a genetic and clinical study was conducted on three families with at least one individual affected by hidrotic ED (20 individuals). We selected the children with molar germs 37 and 47. The eruption axes of these germs were then traced on the panoramic images at the initial time (T_0_) and 1 year later (T_0_ + 1 year), and the deviations between these axes were measured.

**Results:**

A significant familial consanguinity was shown. Eight subjects presented with the hidrotic ED phenotype. Among them, three individuals carried germs 37 and 47. Over time, the measured deviations between the eruption axes of the latter displayed, in the majority of the cases, a distal inclination toward the ramus.

**Limitations:**

A larger sample size is mandatory to assess the frequencies and treatment modalities.

**Conclusion:**

The presence of germs in the lower second permanent molars in patients with hidrotic ED is an important clinical symptom that should be monitored to detect and prevent ectopic migration of these teeth.

**Clinical significance:**

In hidrotic ED cases, the study of the presence of the second lower permanent germs must include clinical and radiological examinations. Establishing an inter-ceptive treatment is necessary to prevent the migration of the molars in question.

**How to cite this article:** Sfeir E, Aboujaoude S. Impacted Lower Second Permanent Molars at the Ramus and Coronoid Process: A New Clinical Symptom of the WNT10A Mutation in Ectodermal Dysplasia. Int J Clin Pediatr Dent 2017;10(4):363-368.

## INTRODUCTION

Ectodermal dysplasia is a rare disease, occurring in 7 in 10,000 births, that presents in more than 200 different clinical forms.^1^ Freire-Maia and Pinheiro^2,3^ described the four major clinical manifestations of ED: Abnormalities in the hair, nails, teeth, and sweat glands.

One particular clinical form is hidrotic ED with the WNT10A gene mutation. This mutation plays an important role in the molecular pathogenesis of nonsyndromic cases of hypodontia, ED, and other rare syndromes, such as odonto-onycho-dermal dysplasia (OODD) and Schopf-Schulz-Passarge syndrome (SSPS).^4-10^

The aim of this study was to identify a new clinical symptom, i.e., specific to hidrotic ED with the WNT10A mutation. It addresses the migratory trend of the lower second permanent molars, when they are present, to the ramus or coronoid process. To the best of our knowledge, no data in the literature describe this trend in the case of hidrotic ED.

## MATERIALS AND METHODS

A 10-year-old male patient (P1) was referred to the Department of Pediatric Dentistry at the Lebanese School of Dentistry, complaining of severe oligodontia. After clinical and radiological examinations, a primary diagnosis of hidrotic ED was established. A treatment plan including prosthesis and implants was implemented.^11^ Informed consent was also obtained from all of the patient’s family members, who underwent clinical, radiological, and genetic examinations to confirm the diagnosis.

**Fig. 1:**
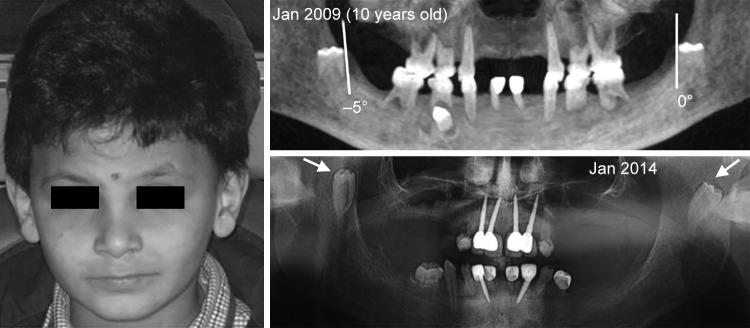
Patient no. 3 (P1) presented with ectopic migration of the lower second permanent molar germs due to the absence of interception. His eyebrow hairs are scattered and sparse with severe oligodontia in permanent dentition

The examination axes that were applied are outlined below:

- *Genealogic Axis:* A three-generation family pedigree was established, taking into account the clinical and radiological examinations and parental consanguinity.
- *Genetic Axis:* Genetic examination was performed on saliva samples from the families (n = 3) with at least one member affected (ORAgene DNA, DNA Genotex Inc. Ottawa, Canada). The genetic examinations aimed to identify the gene involved.
- *Clinical Axis:* An unpredicted panoramic radiography examination on patient P1, 5 years later, showed the migration of the lower second permanent molars (37, 47) to the ramus and the coronoid process respectively (Fig. 1).

Retrospective examinations of the first panoramic radiographs and recall intraoral examinations were performed on all of the subjects who were clinically affected (n = 8). In cases showing the presence of germs 37 and 47 (n = 2), a second panoramic radiograph was conducted 12 months later. This was done to detect any unusual deviations of the axis of eruption that had occurred over time and the tendency of the concerned teeth to migrate to the ramus. The main objective was to establish an interceptive treatment.

**Fig. 2:**
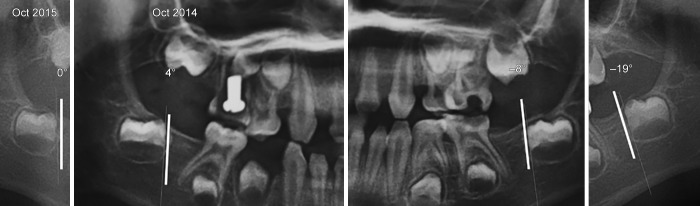
Comparison of the eruption axes of the lower second permanent molar germs after 1 year. A screw is present into the 55

**Fig. 3:**
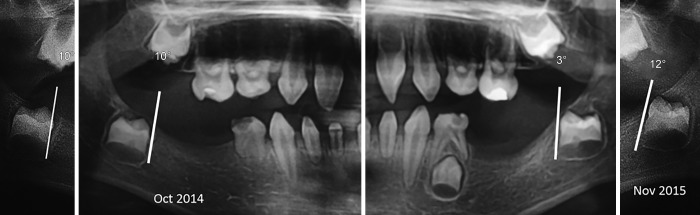
Comparison of the eruption axes of the lower second permanent molar germs after 1 year

In these cases, the axis of eruption was drawn tangential to the mesial surface of each concerned tooth germ (37, 47) on the first and second panoramic X-rays. The angle formed by the tangent to the vertical was measured and recorded on each radiograph (Figs 1 to 3). The deviation and the direction of deviation (mesial or distal) of the axis of eruption were calculated and noted. Similarly, panoramic X-rays were performed on the five other patients who were missing germs 37 and 47, in an attempt to find any new pathological symptoms.

A literature search was initiated to identify clinical cases describing impacted lower second permanent molars in the following situations: (1) Oligodontia, (2) hidrotic ED, (3) hidrotic ED with the WNT10A mutation, and (4) ED with facial phenotypes similar to the one we described. The main objective was to determine the presence or absence of germs 37 and 47 in these patients.

## RESULTS

**Fig. 4:**
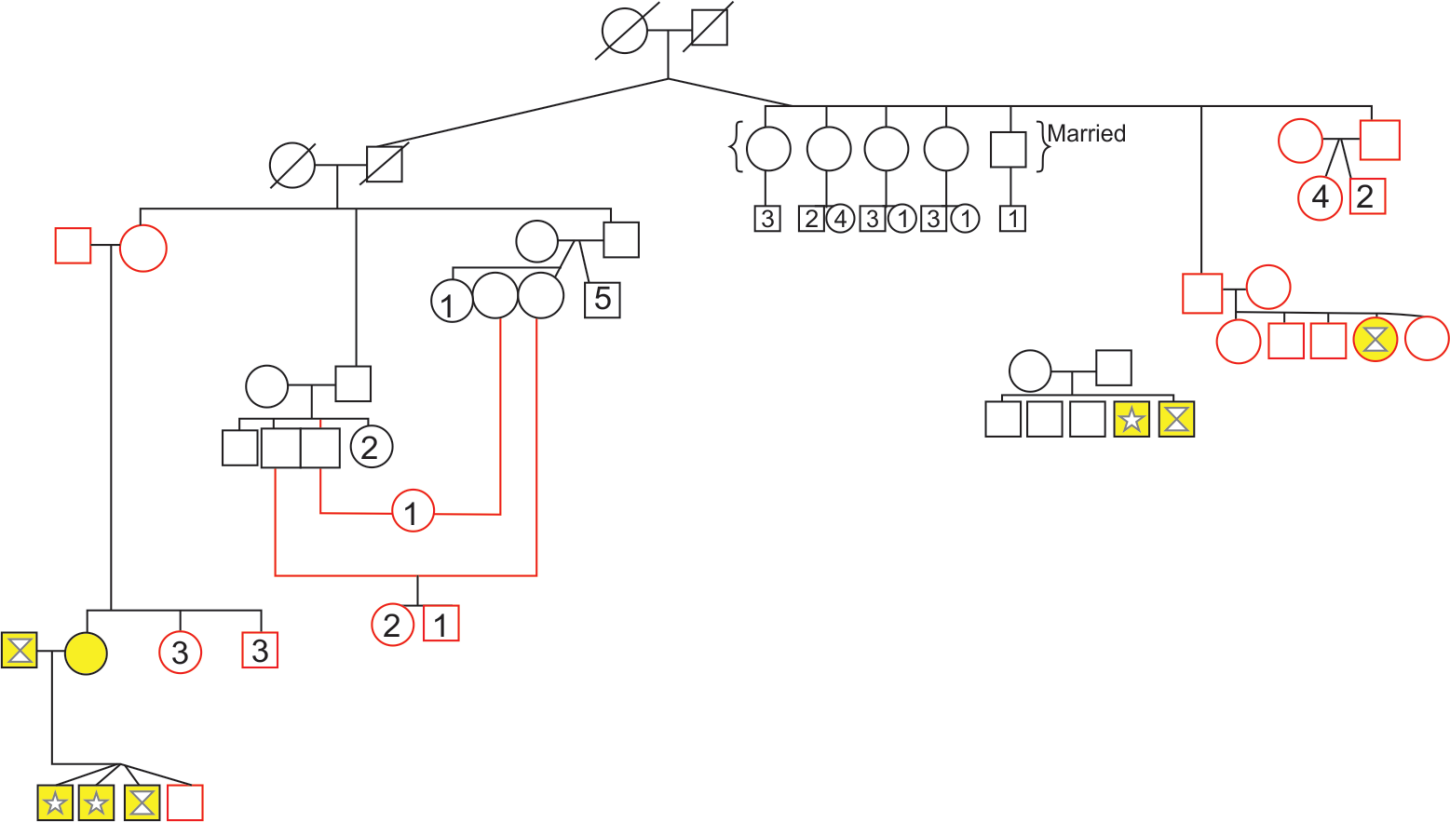
Genealogical pedigree representing the extent of familial consanguinity

The analysis of the family pedigree revealed six consanguineous marriages between seven families. Among three of these families, eight had members with hidrotic ED (Fig. 4).

**Fig. 5:**
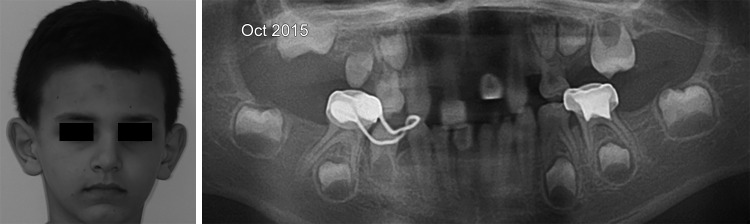
Patient no. 1 after dental treatment. His eyebrow hairs are scattered and sparse with severe oligodontia in permanent dentition

**Fig. 6:**
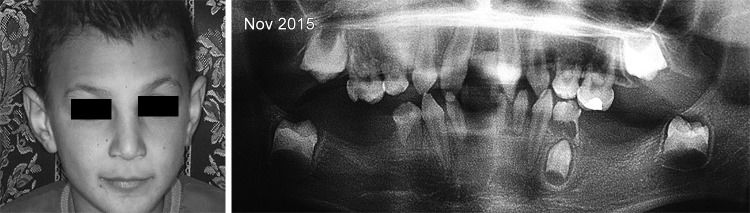
Patient no. 2 has one filling in the second upper left primary molar. His eyebrow hairs are scattered and sparse with severe oligodontia in permanent dentition

The clinical, radiological, and phenotypic analyses produced the following results (Figs 1 to 3, 5 and 6):

- Normal facial phenotypes and subnormal dermatological status (dry skin)

**Fig. 7:**
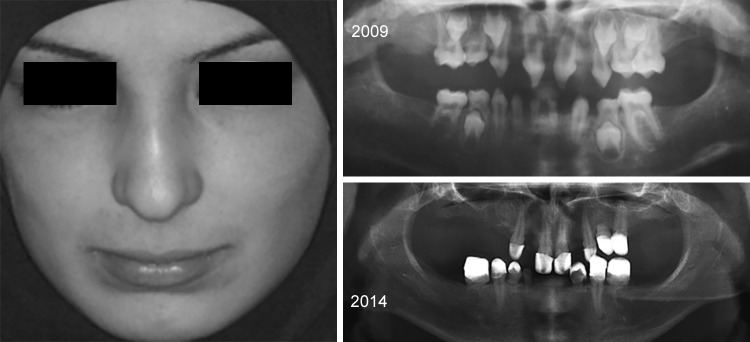
Agenesis of all of the permanent molars in a patient from the same group of families

- Lack of hypotrichosis on the scalp and hair that was dry and dull
- No hypohidrosis
- Scattered and sparse eyebrow hairs (a particular and permanent symptom)
- Thin nails
- Agenesis of two or four primary incisors with a tendency to form a conical shape
- Severe oligodontia in permanent dentition and conical upper incisors (when incisors were present)
- A phenotype generally distinct from that of hypohi-drotic ED

The genetic analyses of the 20 individuals from the three families affected by hidrotic ED confirmed the presence of the WNT10A gene mutation [ChR2: 219755026G> T, NM_025216.2 (WNT10A): c.697G> T, *p.Glu233 (homozygous)]. The analyses were conducted in collaboration with the Center for Dental Manifestations of Rare Diseases, Faculty of Dentistry, University Hospital, Strasbourg, France.

**Table 1:** **Table 1:** A comparison between eruption axes at time T_0 _and T_0_+1 year

*Tooth*				*37*				*47*	
Period		T : 0		T: +1 year		T : 0		T : +1 year	
Patient 1		–8°		–19°		4°		0°	
				Mesial inclination				Distal inclination	
Patient 2		3°		12°		10°		10°	
				Distal inclination				No inclination	
Patient 3 P1		0°		Ectopic migration		–5°		Ectopic migration	

**Table 2:** **Table 2:** Summary of the literature on the presence or agenesis of lower second permanent molar germs in individuals with hidrotic ED or the WNT10A mutation

*Authors*		*Diagnosis*		*Cases: Presence of 37 and 47*		*Cases: Agenesis of 37 and 47*		*Migration of 37 and 47*	
Sfeir et al^11^		ED (no gene identification)		1		1			
Clauss et al^6^		ED and WNT10A mutation				1			
Vink et al^15^		OODD/SSPS/ED WNT10A mutation				5			
He et al^16^		ED and WNT10A mutation				6			
Bohring et al^10^		OODD		2 : 37 and 47 1: only 37		8			
Nawaz et al^17^		WNT10A mutation and OODD				6			
Castori et al^14^		SSPS (no gene identification)				2			
Adaimy et al^9^		WNT10A mutation and OODD				6 (oligodontia) agenesis (37,47) not specified			
Guler et al^12^		ED (no gene identification)						1	
Avcu et al^13^		Severe hypodontia (no gene identification)				1			
Megarbane et al^8^		OODD (no gene identification)				3			
Megarbane et al^7^		ED (no gene identification)				2			

The second X-ray examination indicated that five of the patients had (1) lost most of their temporary teeth and were in the process of losing those that remained (acute root resorption) and (2) agenesis of all permanent molars and in the anterior sectors (Fig. 7). Three other patients had posterior and anterior agenesis, but germs 37 and 47 were present and showed an atypical angle in their axes of eruption (Table 1). In the case of patient no. 1 (Figs 2 and 5), after 1 year, germ 37 inclined 11° mesially and germ 47 inclined 4° distally. In the case of patient no. 2 (Figs 3 and 6), germ 37 inclined 9° distally and germ 47 was unchanged. Finally, in the case of patient no. 3, both germ 37, which was vertical, and germ 47, which inclined 5° distally, migrated to the ramus and coronoid process respectively.

As shown in Table 2, only 5 of the 40 cases examined stated the presence of the lower second permanent molar without any information about their axes of eruption. One (U1) of these five cases showed migration of germs 37 and 47 to the ramus and coronoid process respectively.^6-17^

## DISCUSSION

Implant-retained overdentures and implant-supported constructions for young children with severe oligodontia and anodontia are acceptable methods of dental rehabilitation for children with ED.^18^ The use of mini dental implants, such as prosthetic supports, has resolved the problem of reduced alveolar ridge thickness.^11^

The pedigree revealed the extent of consanguinity, which may explain the presence of the same mutation and phenotype among members of different families. These families subscribe to the same religion and originated from the same region as the families described by Megarbane et al.^7,8^

Many authors have described lower wisdom tooth migration to the ramus and coronoid process.^19,20^ The migration of the lower second permanent molar is exceptional. The only case (U1) reported occurred as a clinical feature of severe oligodontia in patients with hypohidrotic ED.^12^ Although another case of molar migration with severe oligodontia was described, the authors did not specify whether it was an ED or if the migrated teeth were second permanent molars.^13^ In both cases, no genetic investigations for the WNT10A mutation were conducted. The published photo of patient U1 exhibits a similar phenotype to the hidrotic ED cases we describe (i.e., scattered and sparse eyebrow hairs with severe oligodontia in permanent dentition).^12^ This indicates that the patient may have had a case of ED with a WNT10A gene mutation.

The WNT10A mutation has been described in various ED syndromes.^4-6^ In 2007, Adaimy et al^9^ were the first authors to relate a homozygous mutation in the WNT10A gene to OODD and, in 2009, Bohring et al^10^ were the first to describe SSPS in patients with WNT10A mutations.

According to the examinations conducted on patient P1 and the panoramic radiographic analyses of the studied cases, the following assertions can be made: (1) Any vertical axis or distal inclination of the axis of eruption of the lower second permanent molar after 1 year is a possible atypical migration or retention of the concerned tooth and (2) maintaining the same mesial inclination or an increase in the mesial inclination of the axis of eruption after 1 year is a situation that warrants monitoring until the tooth emerges into the oral cavity.

In general, when a tooth has an ectopic axis of eruption, it is imperative to interfere surgically to restore the tooth’s axis or facilitate its emergence.^21^ When a molar distal inclination is confirmed, an interceptive surgical procedure must be performed. Furthermore, periodic and thorough follow-ups are recommended for teeth with vertical axes and, consequently, intervention is required at the first sign of distal inclination of their axes of eruption. The case of no. 3 (P1) illustrates the distal inclination of a previously vertical axis before eruption that leads to the migration of molars to the ramus (Fig. 1).

These findings are of great interest, as the phenomenon of ectopic migration to the ramus or the coronoid process occurred in hidrotic ED patients with a WNT10A mutation. To the authors’ knowledge, no such cases have been described in the literature.

Finally, we believe that this anomaly is a new clinical symptom of hidrotic ED with the WNT10A gene mutation. When the lower second permanent molars are present, close monitoring of their axes of eruption is required to detect and prevent ectopic migration.

Hidrotic ED is a very rare disease. As this study included a limited number of cases, further investigations using larger sample sizes are required. Future studies must assess the frequencies and treatment modalities for these clinical cases.

## CONCLUSION

Hidrotic ED accompanied by the mutation of the WNT10A gene presents with phenotypic dentofacial variability. The presence of lower second permanent molars in these individuals should be a particular clinical symptom to add to the overall clinical features of hidrotic ED and requires careful and regular monitoring to detect and prevent ectopic migration of these teeth to the ramus or coronoid process.

### Why is This Paper Important to Pediatric Dentists?

Pediatric dentists should be aware of the possible migration of the lower second permanent molars in hidrotic ED cases. In severe oligodontia cases, pediatric dentists should be able to differentiate between hypohidrotic and hidrotic ED.

### What does This Paper contribute?

Considering the importance of tooth preservation in hidrotic ED cases, the early detection of the lower second permanent molar germs helps to maximize treatment and facilitate their appropriate positioning on the dental arch. Distal inclination of the lower second permanent molar germs’ eruption axes should be considered an alarming sign indicating the possible ectopic migration of these teeth.
